# UPLC-Q-TOF/MS-Based Plasma Metabolomics to Evaluate the Effects of Aspirin Eugenol Ester on Blood Stasis in Rats

**DOI:** 10.3390/molecules24132380

**Published:** 2019-06-27

**Authors:** Dongshuai Shen, Ning Ma, Yajun Yang, Xiwang Liu, Zhe Qin, Shihong Li, Zenghua Jiao, Xiaojun Kong, Jianyong Li

**Affiliations:** 1 Key Lab of New Animal Drug Project of Gansu Province; Key Lab of Veterinary Pharmaceutical Development, Ministry of Agriculture; Lanzhou Institute of Husbandry and Pharmaceutical Science of Chinese Academy of Agricultural Sciences, Lanzhou 730050, China; 2 College of Veterinary Medicine, Agricultural University of Hebei, Baoding 071000, China

**Keywords:** blood stasis, AEE, aspirin, metabolomics, UPLC-Q-TOF/MS

## Abstract

Aspirin eugenol ester (AEE) is a novel compound that is formed from the esterification of aspirin (acetylsalicylic acid (ASA)) and eugenol. This study aimed to investigate the effects of AEE on blood stasis in rats and to characterize the underlying mechanisms using a plasma metabolomic study. The results indicate that AEE and ASA could modulate whole blood viscosity (WBV), plasma viscosity (PV), blood coagulation parameters, platelet count, platelet aggregation, lactate dehydrogenase (LDH), creatinine (CR) and the levels of thromboxane A2 (TXA_2_) and 6-keto prostaglandin F1α (6-keto-PGF_1α_). The metabolic profiles of the plasma samples from all groups were clearly separated in the score plots. Nineteen potential metabolites were selected and identified, and disordered levels of these metabolites could be regulated by AEE and ASA. Pathway analysis showed that the mechanism of action of AEE on blood stasis might be principally related to the metabolism of amino acid, fatty acid, energy and glycerophospholipid. The above results indicate that AEE protected the rats against blood stasis, and that this effect might have been caused by the anticoagulation activity of AEE and its abilities to maintain a balance between TXA_2_ and PGI_2_, reduce blood viscosity, inhibit platelet aggregation and normalize the plasma metabolic profile.

## 1. Introduction

Blood stasis is one of the most common clinical syndromes and is characterized as a pathological state of blood stagnation, delayed and impeded blood flow and local stagnation and blockade of blood vessels [1]. Hemorheological disorders have been recognized as pathological mechanisms and have diagnostic criteria of blood stasis that also include abnormalities of blood viscosity, blood coagulation function and platelet aggregation [2]. Modern medical research indicates that blood stasis is the common pathological basis of various cardiovascular diseases (CVDs), such as thrombosis [3], hyperlipidaemia [4], cerebrovascular disease [5], hypertension [6] and coronary heart disease [7]. CVDs are the leading global disease burden for both mortality and morbidity. The principle of treating CVDs by improving blood circulation and decreasing blood stasis has received increased attention in recent years [8].

Aspirin (acetylsalicylic acid (ASA)) is extensively used for the treatment of inflammation, fever, pain and cardiovascular disease. However, gastrointestinal damage caused by ASA largely limits its long-term use [9]. Eugenol, the active ingredient in clove and clove oil, is a safe essential oil and possesses many therapeutic effects such as anti-inflammation, anticoagulation, antioxidation and anti-platelet aggregation [10,11,12]. However, eugenol is difficult to use in a clinical setting because it is an irritant and is vulnerable to oxidation, since it contains a phenolic hydroxyl group. To solve the limitations presented by both ASA and eugenol, the starting precursor aspirin was reacted with SOCl_2_ to generate an acyl chloride compound via esterifying eugenol to aspirin eugenol ester (AEE) [13], followed by masking the phenolic hydroxyl group of eugenol and the carboxyl group of ASA. Therefore, AEE could improve structural stability and reduce gastrointestinal side effects [14]. Currently, the teratogenicity, metabolism, acute and sub chronic toxicity and pharmacodynamics of AEE have been estimated, and the relative results indicate that AEE is a safe and promising drug candidate [14,15,16,17,18].

As a branch of systems biology, metabolomics approaches can be used to explore metabolic patterns through a comprehensive investigation of the metabolite changes in living systems [19]. Metabolomics has been increasingly applied in the fields of drug safety, pharmacodynamic evaluations and disease biomarker discovery [20]. Due to its decreased analytical time and increased sensitivity, ultra-performance liquid chromatography (UPLC) coupled with mass spectrometry (MS) is employed in many metabolomics studies [21]. Recently, MS-based metabolomics has been successfully performed in many cases to evaluate the therapeutic effects of drugs [22]. For example, the plasma and urinary metabolic profiles and drug effects of many traditional Chinese medicines, such as Taohong Siwu Decoction and Xindi soft capsules, have been investigated in rats with blood stasis [23,24].

ASA is widely used to treat blood stasis by promoting blood circulation [25]. A previous study indicated that AEE possess antithrombotic activity by improving the hemorheological events in a rat tail thrombosis model [17]. However, the mechanism of AEE on blood stasis is still unclear, and there has been no report of a metabolomic study of AEE for blood stasis. In this study, the effects of AEE on acute blood stasis (ABS) were evaluated in rats by measuring blood viscosity, plasma coagulation parameters, blood count, blood biochemistry and the levels of thromboxane B_2_ (TXB_2_) and 6-keto-PGF_1α_. Furthermore, a plasma metabolomic approach using UPLC-Q-TOF/MS was performed to assess the integral effect of AEE on ABS in rats. Moreover, we also compared the effects of AEE and ASA on treating blood stasis, which could illustrate the differences of AEE over ASA.

## 2. Results

### 2.1. Effects of AEE on WBV and PV

**Figure 1:**
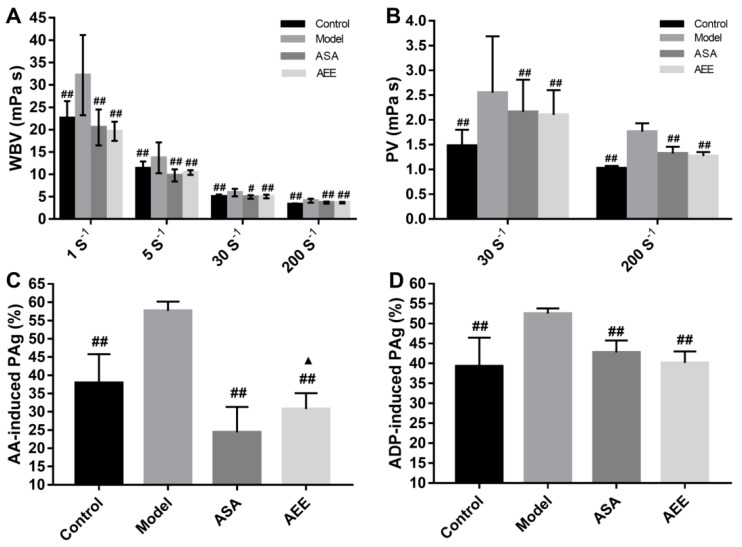
Results of blood viscosity and platelet aggregation in different groups. (**A**,**B**) Effects of AEE on WBV and PV. (**C**,**D**) Effects of AEE on AA and ADP-induced platelet aggregation. ASA: aspirin; AEE: aspirin eugenol ester; WBV: whole blood viscosity; PV: plasma viscosity; PAg: platelet aggregation. Compared with the model group, ^#^ *p* < 0.05, ^##^ *p* < 0.01; compared with the ASA group, ^▲^ *p* < 0.05.

Whole blood viscosity (WBV) and plasma viscosity (PV) are important parameters to estimate the success of blood stasis model. As shown in Figure 1A and B, when compared with the control group, the WBV was markedly increased at all shear rates (1, 5, 30 and 200 s^−1^) in the model group (*p* < 0.01). The PV was also significantly increased in the model group at shear rates of 30 and 200 s^−1^ compared with the control group (*p* < 0.01). These results indicate that the blood stasis model was successfully established in this study. Both ASA and AEE could significantly decrease the WBV (*p* < 0.05 or *p* < 0.01) at all shear rates. At low or high shear rates (30 and 200 s^−1^), the PV was significantly reduced by treatment with AEE and ASA (*p* < 0.01). There was no significant difference in the WBV or PV between the ASA and AEE groups.

### 2.2. Effects of AEE on Coagulation Parameters and Platelet Aggregation

In comparison with the control group, the fibrinogen (FIB) content was significantly increased (Table 1, *p* < 0.01) but the TT was markedly decreased in the model group (*p* < 0.01). When compared with the model group, both ASA and AEE significantly prolonged the prothrombin time (PT) and thrombin time (TT) (*p* < 0.05 or *p* < 0.01), but reduced the FIB content (*p* < 0.05). Additionally, the FIB level was found to be lower in the AEE group than in the ASA group (*p* < 0.05).

As shown in Figure 1C,D, adenosine diphosphate (ADP)- and arachidonic acid (AA)-induced platelet aggregation were significantly increased in the rats with blood stasis compared with the control (*p* < 0.01). Both ASA and AEE inhibited platelet aggregation. ASA had a better inhibitory effect than AEE in AA-induced platelet aggregation. With regards to ADP-induced platelet aggregation, there was no significant difference between the ASA and AEE groups.

### 2.3. Effects of AEE on Haematological Analysis and Biochemistry Parameters

As shown in Table 2A, the white blood cell (WBC), monocyte count (MONON) and neutrophilic granulocyte (Gran) values were markedly increased in the model group when compared with the control group (*p* < 0.01), which might have been caused by inflammation in the ABS rats. Additionally, the percentage of platelet (PCT) and platelet (PLT) levels in the model group were markedly decreased compared to those of the control group (*p* < 0.01). In comparison with the model group, ASA and AEE significantly increased the PLT and PCT levels (*p* < 0.01). Notably, the AEE group had a markedly higher PCT level than the ASA group (*p* < 0.05).

When compared with the control group, the albumin (ALB), alkaline phosphatase (ALP), ALB/ globulin (GLB), and glucose (GLU) values were significantly reduced in the model group (Table 2B), while the alanine transaminase (ALT), aspartate aminotransferase (AST), ALT/AST, lactate dehydrogenase (LDH), blood urea nitrogen (BUN) and creatinine (CR) values were markedly elevated (*p* < 0.05 or *p* < 0.01). Treatment with ASA and AEE markedly increased ALT, AST, creatine kinase (CK), BUN and triglyceride (TG) and reduced total bilirubin (T-BIL), total protein (TP), ALB, globulin (GLB), ALP and GLU (*p* < 0.05 or *p* < 0.01). In addition, compared with the model group, uric acid (UA) and CR were elevated in the ASA and AEE groups, respectively. Distinct differences were observed between the CR and UA values of the ASA and AEE groups (*p* < 0.05).

### 2.4. Measurement of Plasma TXB_2_ and 6-keto-PGF_1α_

**Figure 2:**
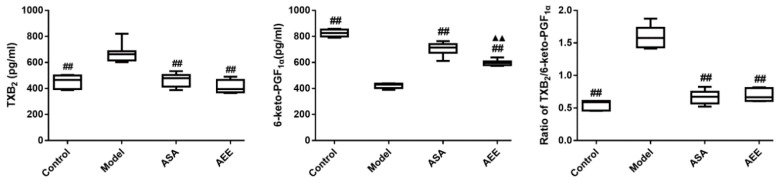
Effects of AEE on TXB_2_ and 6-keto-PGF_1α_ levels. TXB_2_: thromboxane B_2_. Compared with the model group, ^##^ *p* < 0.01; compared with the ASA group, ^▲▲^ *p* < 0.01.

TXB_2_ levels were significantly elevated in the model group compared with those of the control group, whereas 6-keto-PGF_1α_ was markedly decreased (*p* < 0.01, Figure 2). In comparison with the model group, 6-keto-PGF_1α_ was markedly increased, while TXB_2_ was reduced in both the ASA and AEE groups (*p* < 0.01). Additionally, TXB_2_/6-keto-PGF_1α_ ratios were calculated for the different groups. The results indicate that this ratio was markedly higher in the model group than in the control group (*p* < 0.01). Treatment with ASA and AEE significantly reduced the TXB_2_/6-keto-PGF_1α_ ratio. Compared with ASA, AEE could significantly lower 6-keto-PGF_1α_ (*p* < 0.01), while no remarkable differences in TXB_2_ level or the TXB_2_/6-keto-PGF_1α_ ratio were observed between the ASA and AEE groups.

### 2.5. Effect of AEE on Plasma Metabolomics

Total ion chromatograms (TICs) of the plasma are shown in Figure S1. Under the present experimental conditions, the TIC showed good peak shape, intensity and separation, which indicated that the chromatographic and MS conditions optimized in present study were suitable for the plasma analysis. Unsupervised PCA was performed on all of the samples in the study. In the positive and negative modes, the results showed that 45.2% and 41.4% of the total variance was elucidated by the first two principal components, respectively (Figure S2). The distributions of the quality control (QC) samples in the PCA score plots are shown in Figure S2. The QC samples were found to be clustered tightly in both modes of the PCA score plots, which indicated that the analytical method was robust and had good repeatability and stability. In the score plots, the samples from the four different groups showed clear separation and revealed an obvious distinction in the plasma metabolic profiles of the rats.

**Figure 3:**
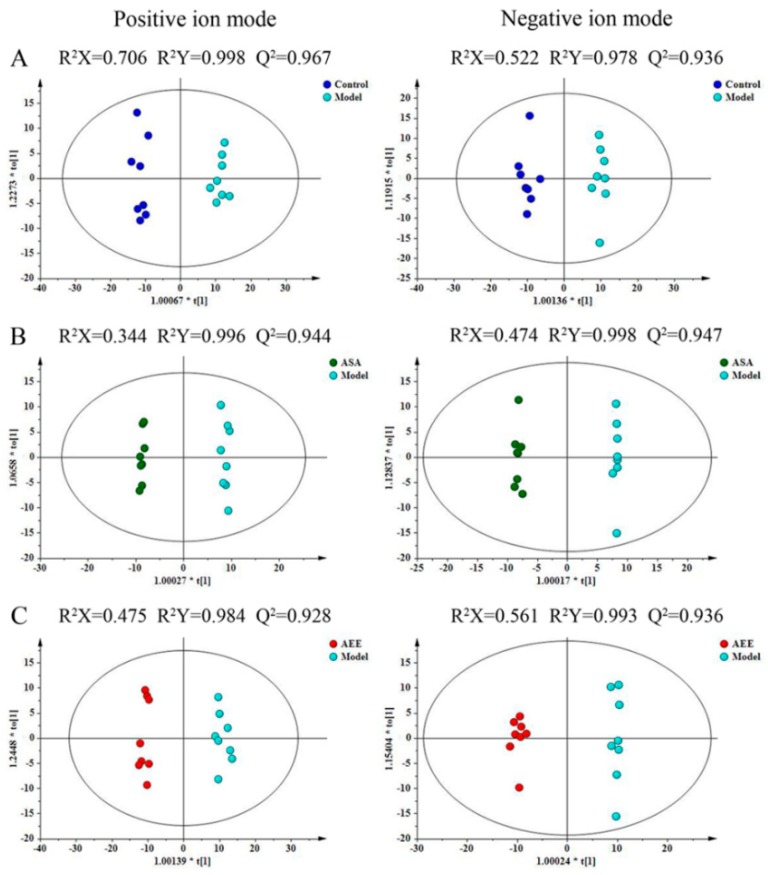
The effect of AEE on the metabolomic profile of plasma from rats with blood stasis. The OPLS-DA score plots of different groups in positive and negative modes. (**A**) Control group versus model group, ESI+: R^2^X = 0.706, R^2^Y = 0.998 and Q^2^ = 0.967; ESI-: R^2^X = 0.522, R^2^Y = 0.978 and Q^2^ = 0.936. (**B**) ASA group versus model group, ESI+: R^2^X = 0.344, R^2^Y = 0.996 and Q^2^ = 0.944; ESI-: R^2^X = 0.363, R^2^Y = 0.995 and Q^2^ = 0.93. (**C**) AEE group versus model group, ESI+: R^2^X = 0.475, R^2^Y = 0.984 and Q^2^ = 0.928; ESI-: R^2^X = 0.494, R^2^Y = 0.976 and Q^2^ = 0.912.

To further maximize the separation and identify the metabolites, a supervised orthogonal partial least squares discriminant analysis (OPLS-DA) approach was used. OPLS-DA models were subsequently established between the model and other groups to enhance the variation. The OPLS-DA score plots presented an obvious separation between the model and other groups without any overlap in either the positive or negative modes (Figure 3), which indicated that there was a perturbation of the plasma metabolic profile in the model group and that treatment with both ASA and AEE could cause substantial alterations in the profile compared to that of the model group. As shown in Figure S3, clear separation between AEE and ASA groups could be observed, indicating that there was a difference in the plasma metabolite profiles from the two groups. In addition, the OPLS-DA results were in accordance with the PCA score plots. The R^2^X, R^2^Y and Q^2^ values were at least 0.344, 0.978 and 0.928 in the OPLS-DA models, respectively, which suggests that the models were robust and had predictive abilities.

### 2.6. Identification of Potential Biomarkers

S-plot, variance importance for projection (VIP) and *p*-values were employed to identify the metabolites that had an important influence on the group separation. S-plots of the plasma samples are shown in Figure S4. With VIP values above 1.0 and *p*-values less than 0.05, 19 metabolites in the plasma were considered as potential metabolites that were correlated with blood stasis and drug effects (Table 3). Metabolite information including molecular formula, compound name, corresponding concentration fold changes and metabolic pathways are also shown in Table 3. Twelve metabolites showed a marked increase and seven metabolites were significantly reduced in the model group in comparison with the control. The changes in the relative content of these metabolites that were induced by ABS were normalized by treatment with AEE and ASA to various degrees.

Hierarchical clustering analysis was performed to display the relationships and differences among metabolites and samples. The heatmap that was generated with MetaboAnalyst calculated the distance by Person and the clustering by Ward. As shown in Figure S5, metabolites in the same pathway or with similar abundance patterns were positioned closer together. For example, seven LysoPC metabolites that resulted from glycerophospholipid metabolism were clustered together; amino acids such as tryptophan, isoleucine and valine were also clustered together. Additionally, the dendrogram in the vertical axis of Figure S5 displayed a clustering of samples from different groups. Notably, samples in the control and model groups were separated on the different branches, indicating the ABS model was successfully established. Furthermore, the samples in the AEE group were clustered together near those in the control group, suggesting that the abundance pattern of the metabolites in the AEE and control groups was similar. In addition, a statistical analysis was performed among the control, AEE and ASA groups (Table S1). AEE was found to have a better regulating effect than ASA on some metabolites such as LysoPC (15:0), LysoPC (16:0), LysoPC (18:2), palmitoylcarnitine and phenylalanine. These results suggested that AEE treatment regulated the metabolites to near-normal levels to reverse the effect of ABS, which had better efficiency than ASA.

### 2.7. Pathway Analysis

**Figure 4:**
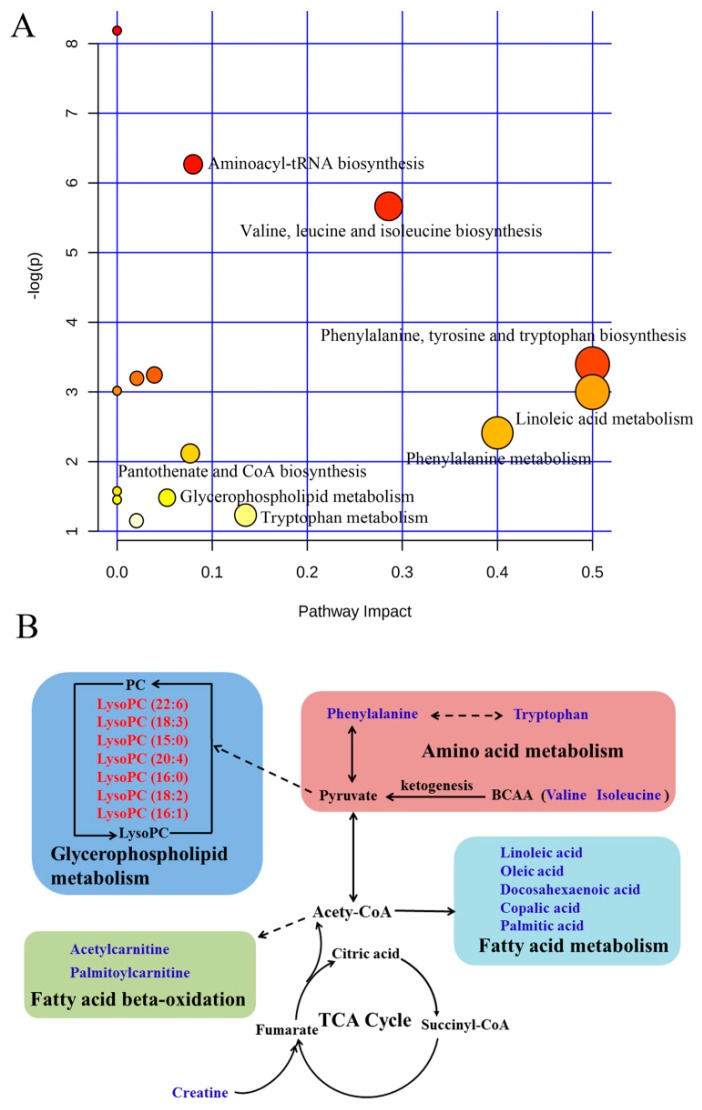
Disturbed pathways in response to blood stasis and AEE treatment. (**A**) Summary of pathway analysis from MetaboAnalyst. All of the matched pathways are displayed as circles. The color and size of each circle are based on its -log (p) value and pathway impact value, respectively. The x-axis represents the pathway impact value that was calculated from the pathway topological analysis, and the y-axis represents the -log (p) value that was obtained from the pathway enrichment analysis. The metabolic pathway with a high pathway impact value and -log (p) value shows that the identified metabolites had a key position and influence on this pathway. (**B**) The potential metabolic network that was disrupted in rats with blood stasis and was altered by AEE treatment.

Different metabolites were analyzed by MetaboAnalyst software to reveal their association with metabolic pathways. Relative pathways with an impact-value greater than 0.05 were identified as potential target pathways (Table S2). The pathway analysis results are shown in Figure 4A, and they revealed that the primary pathways that responded to ABS were aminoacyl-tRNA biosynthesis, linoleic acid metabolism, phenylalanine, tyrosine and tryptophan biosynthesis, phenylalanine metabolism, valine, leucine and isoleucine biosynthesis, tryptophan metabolism, pantothenate and CoA biosynthesis and glycerophospholipid metabolism.

## 3. Discussion

Blood stasis is closely related to hemorheological disorders, which result in abnormalities in WBV, PV, blood coagulation parameters and platelet aggregation. In the present study, the increased WBV, PV and FIB and the reduced TT in the model group suggested the successful establishment of ABS in the rats. AEE and ASA could markedly reduce WBV, PV, FIB and platelet aggregation, and increase PT and TT, indicating that both ASA and AEE might have therapeutic effects on ABS in rats.

WBV, which corresponds to the apparent viscosity, is influenced by platelets, plasma and red blood cells (RBCs) and refers to the intrinsic resistance of the blood flow in blood vessels [26,27]. In this study, the increased WBV at all shear rates in the model group suggested that there was a hyper-viscosity of the blood in the rats with blood stasis. Treatment with AEE markedly reduced the WBV, which indicated that the intrinsic resistance of the blood flow was decreased. WBV is partly determined by PV. AEE treatment was also found to prevent the increase of PV in ABS rats, indicating that the reduction of WBV was partly attributed to the decrease of PV. PV is dependent on the types and concentrations of proteins that are in the plasma [2]. FIB is an important plasma protein and was significantly reduced by AEE treatment, which might be one of reasons for the reduction in PV. There was no difference in WBV or PV between the ASA and AEE treatment groups, suggesting that equimolar ASA and AEE treatments had similar effects on blood viscosity. It is found that the abnormal blood viscosity firstly emerges in blood stasis, followed by circulatory complications, thrombosis and the narrowing of blood vessels, which indicates that blood viscosity disorder is the pathological basis of blood stasis. In high blood viscosity, an increase of FIB concentration and an imbalance of TXA_2_ and 6-keto-PGF_1α_ can accelerate the platelet aggregation rate, causing thrombosis and blood vessel injury. In this study, AEE and ASA significantly decreased blood and plasma viscosity to damage the development foundation of the blood stasis, which benefited the improvement of FIB formation, platelet aggregation and the balance of TXA_2_ and 6-keto-PGF_1α_.

PT reflects the overall efficiency of the extrinsic clotting pathway (exogenous blood coagulation system) [28], while FIB and TT reflect the third coagulation phase in plasma [29]. Either an extension of TT or a decrease in FIB indicate a suppression of thrombin-mediated fibrin formation. Treatment with AEE markedly prolonged the PT and TT and decreased the FIB in comparison with the model group, showing the inhibitory effect of AEE on coagulation pathways and fibrin formation. Notably, the mean level of FIB in the AEE group was lower than that in the ASA group, which might suggest that AEE had a stronger inhibitory effect than ASA on FIB formation.

Platelets link to each other through the binding of the integrin receptor on platelets to soluble plasma FIB; therefore, plasma FIB is an essential material for platelet aggregation [30]. In this study, the reduced FIB content in the ASA and AEE groups contributed to the inhibition on platelet aggregation. Previous studies have indicated that the platelet aggregation level is higher in ABS rats [2]. With the increased platelet aggregation level, many platelets become activated and then aggregate into thrombin, followed by a decrease in the amount and percentage of platelets in circulating blood, resulting in significantly reduced PLT and PCT levels in the model group. Saeed et al. reported that eugenol could inhibit platelet-activating factor-induced platelet aggregation and arachidonic acid metabolism via the cyclooxygenase and lipoxygenase pathways in human platelets [12], which might be one of the reasons for the inhibitory effects of AEE on platelet aggregation. Moreover, platelet aggregation is thought to be one of the factors that determine blood viscosity [31]; therefore, a reduction in WBV and PV could be ascribed to a decreased platelet aggregation level in the AEE and ASA groups.

TXA_2_ is a typical platelet aggregation agonist and can induce vasospasm, platelet aggregation and thrombus formation, whereas PGI_2_ may prevent platelet aggregation and possess the vasodilator action [32]. TXA_2_ and PGI_2_ are involved in CVDs, as they play a crucial part in homeostasis [33]. A pathological imbalance between TXA_2_ and PGI_2_ can result in platelet aggregation, vasospasm and thrombosis, followed by the initiation of various types of CVD [34,35,36]. Therefore, the dynamic equilibrium of TXA_2_/PGI_2_ is an essential factor for regulating regional flow and vessel wall morphology [37]. The stable metabolites of TXA_2_ and PGI_2_ are TXB_2_ and 6-keto-PGF_1α_, respectively. In this study, AEE could markedly upregulate 6-keto-PGF_1α_ and downregulate TXB_2_ levels in the serum of ABS rat, indicating that AEE promoted blood flow and removed blood stasis by regulating active substances in the vascular endothelium.

LDH and CR are indicators of liver and kidney function. The liver and kidney are two of the major organs that participate in the metabolic process. Abnormal blood circulation caused by blood stasis decreased the rates of metabolite elimination, and the accumulation of metabolic waste in the liver and kidney finally resulted in injury to the liver and kidney that was verified by the increase in LDH and CR in the model group [38]. Unexpectedly, compared to the model group, ALT and AST levels were increased, while the AST/ALT ratio was significantly reduced in the ASA and AEE groups. ALT and AST are released from hepatic cells, and it is speculated that AEE and ASA may influence physiological and biochemical function and the integrity of hepatocytes, leading to an increase of ALT and AST. To some degree, decreased levels of LDH and CR in the AEE group were beneficial to improve blood stasis in rats. Notably, more studies are needed to investigate the effects of AEE on liver and kidney functions.

In this study, 19 potential metabolites were found to be related to ABS and the regulation of AEE treatment. The metabolic metabolite networks were constructed to understand the potentially therapeutic effects of AEE on ABS (Figure 4B). The dysregulated metabolic pathway that was induced by ABS was mediated by AEE treatment, and the therapeutic effects of AEE on ABS were primarily associated with the metabolism of amino acid, fatty acid, energy and glycerophospholipid.

Plasmatic concentrations of phenylalanine, isoleucine, valine and tryptophan were higher in rats with ABS than those in the healthy group, which indicates that the amino acid metabolic pathways were affected in ABS rats. With the application of a ^1^H NMR-based plasma metabolomic approach, Zou et al. found that many essential amino acids, such as isoleucine, valine and lysine, were significantly increased in rats with blood stasis [39], which is partly consistent with our findings. In the present study, rats were placed in ice-cold water to induce ABS. Previous studies have indicated that exposure to cold temperatures can influence antioxidant balance, and that increased oxidation can cause protein misfolding [39,40]. Misfolded proteins can be degraded by the proteasome and thus may elevate amino acid levels. Therefore, the elevated levels of amino acids might result from misfolded protein degradation that was induced by cold exposure. As branched chain amino acids, valine and isoleucine are important energy sources of ketogenesis. Elevated levels of valine and isoleucine in the ABS rats indicated that there was an increase of ketogenesis that was related to an impaired energy metabolism [41]. Phenylalanine is biologically converted into tyrosine, which is a precursor of catecholamines such as dopamine, adrenaline and norepinephrine. Increased phenylalanine in plasma might suggest that the production of catecholamines was upregulated, which could thus cause vasoconstriction and affect blood flow. AEE treatment showed a favorable inhibition on the increase of phenylalanine, isoleucine, valine and tryptophan, suggesting that the protective effects of AEE on stasis might be ascribed to improved amino acid metabolism.

It has been proven that there is a close relationship between blood stasis and glycerophospholipid metabolism [42]. In this study, decreased levels of the LysoPCs involved in glycerophospholipid metabolism were observed in the plasma of the ABS rats, and this finding is consistent with the results of previous studies [23]. The changes in these metabolites suggests that glycerophospholipid metabolism was impaired during the pathogenesis of blood stasis. LysoPCs are related to the progression of inflammatory and cardiovascular diseases. John et al. reported that a low vascular concentration of LysoPC could induce platelet aggregation with local tissue ischaemia, perivascular inflammation and vascular damage [43]. The reduction in LysoPCs was inhibited by treatment with AEE, suggesting that the protective efficacy of AEE on blood stasis might be ascribed to its regulatory effects on glycerophospholipid metabolism. AEE was more effective than ASA in regulating LysoPCs; for example, the levels of LysoPC (15:0), LysoPC (16:0) and LysoPC (18:2) in the AEE group were closer to those of the control group than those in the ASA group (Table S1). There was a difference between AEE and ASA on glycerophospholipid metabolism, and this difference might be caused by the synergistic action of salicylic acid and eugenol after the decomposition of AEE in ABS rats.

In this study, linoleic acid, oleic acid, docosahexaenoic acid, copalic acid and palmitic acid, which are all involved in fatty acid metabolism, were increased in the plasma of the rats with blood stasis. As a common saturated fatty acid, palmitic acid plays an important role in fatty acid metabolism and energy production [44]. It was speculated that higher levels of palmitic acid were produced in the model group to increase energy supply. Zhang et al. reported that linoleic acid was significantly increased in the urine of rats with acute blood stasis, which is consistent with our results [23]. Some studies have shown that linoleic acid increased the production of interleukin-8 by vascular smooth muscle cells under conditions of oxidative stress [45]. As the precursor to arachidonic acid, linoleic acid and its metabolites could mediate inflammation. The positive relationship between inflammation and blood stasis is well-known [46]. Therefore, the elevated levels of linoleic acid in the model group might be related to the increased inflammation induced by ABS. A previous study reported that the plasma oleic acid content was increased in patients with coronary heart disease with blood stasis, which agrees with the oleic acid results in the present study [47]. Linoleic acid, oleic acid and docosahexaenoic acid are unsaturated fatty acids. Many experiments have shown that unsaturated fatty acids have an antioxidation effect that can increase superoxide dismutase (SOD) and the activities of catalase and antioxidant enzymes [48]. In contrast to the control rats, the increased oleic acid and docosahexaenoic acid in the plasma of the model rats suggests that increased levels of antioxidants were possibly produced to defend against increasing oxidative stress during the development of blood stasis. In addition, previous studies have indicated that LysoPCs could be degraded into free fatty acids and glycerophosphocholine in solid tumor cells [49]. In the present study, the reduction of LysoPC in plasma might be one of the reasons for the increase of fatty acids such as linoleic acid and oleic acid in the ABS rats. These increases in fatty acids such as linoleic acid, oleic acid and docosahexaenoic acid were significantly inhibited by AEE treatment, indicating that the disrupted fatty acid metabolism was normalized after treatment. The regulatory effects of AEE on these fatty acids might be related to its ameliorative effects on energy metabolism, inflammation and oxidative damage.

Creatine is an energy supplement in tissues and has an important role in energy metabolism [50]. Creatine can facilitate the recycling of adenosine triphosphate (ATP) by converting adenosine diphosphate (ADP) back to ATP via the donation of phosphate groups [51]. A previous study revealed that the suppression of tricarboxylic acid cycle (TCA) activity caused disordered energy metabolism in rats with blood stasis [39]. An increased level of creatine was observed in the rats with blood stasis when compared to the rats in the control group, which suggests that the accumulation of creatine in the plasma was increased to supply an alternative energy source [52]. Palmitoylcarnitine and acetylcarnitine are derivative esters of carnitine, and increased levels of palmitoylcarnitine and acetylcarnitine were observed in the model group, which might suggest that there was an increase in the fatty acid oxidation pathway to provide the required energy. The levels of palmitoylcarnitine and acetylcarnitine were significantly recovered in the AEE group, suggesting that AEE treatment could ameliorate the disturbed energy metabolism to satisfy the energy requirement. Levels of creatine, palmitoylcarnitine and acetylcarnitine were regulated to different degrees after ASA and AEE treatment, which indicates that AEE and ASA had different mechanisms of action on energy metabolism. There is a close relationship between mitochondrial functional damage and energy metabolism. Some studies have indicated that ATP synthase is affected by isotope effects and water viscosity inside mitochondria [53,54]. Reduced plasma viscosity in ASA and AEE groups might be conducive to the improvement of mitochondrial functions. Further studies are needed to investigate the effects of AEE on ATP content and the activities of mitochondrial respiratory chain complexes, which can be assessed by enzyme-linked immunosorbent assay (ELISA) in blood cells.

It is important to note that this study had some limitations. First, this study did not evaluate the effects of AEE on inflammation in ABS rats. It is well-known that blood stasis is related to inflammation, and thus the effects of AEE on inflammatory factors and signaling pathways will be investigated in future studies. Second, quantitative and targeted studies are needed to confirm potential metabolites through a different platform such as NMR or GC-MS. In addition, the underlying mechanism of AEE on metabolic pathways in rats with blood stasis should be explored with additional studies.

## 4. Materials and Methods

### 4.1. Reagents

AEE was synthesized and purified at the Lanzhou Institute of Husbandry and Pharmaceutical Sciences of CAAS [13]. Adrenaline hydrochloride (Adr) was purchased from Tianjin Jinyao Amino Acid Co., Ltd. (Tianjin, China). Carboxymethylcellulose sodium (CMC-Na), ASA and eugenol were supplied from Aladdin Chemical (Shanghai, China). Formic acid and acetonitrile (MS-grade) were purchased from TCI (Shanghai, China) and Thermo Fisher Scientific (Waltham, MA, USA), respectively. The total bilirubin (T-BIL), total protein (TP), albumin (ALB), globulin (GLB), alanine transaminase (ALT), aspartate aminotransferase (AST), alkaline phosphatase (ALP), lactate dehydrogenase (LDH), blood urea nitrogen (BUN), creatinine (CR), glucose (GLU), creatine kinase (CK), uric acid (UA) and triglyceride (TG) kits for the blood biochemical analysis were provided by Ningbo Medical System Biotechnology Co., Ltd. (Ningbo, China). The TXB_2_ and 6-keto-PGF_1α_ ELISA kits were obtained from Cayman Chemical (Ann Arbor, MI, USA).

### 4.2. Animals and Grouping

Thirty-two female Wistar rats (license approval No. SCXK (Gan) 2013–0002) with body weights of 220–240 g at seven weeks of age were provided by Lanzhou University (Lanzhou, China). The rats were maintained in specific pathogen-free (SPF) conditions at a constant temperature of 22 ± 2 °C with a relative humidity of 50 ± 5%. The rats were acclimated for two weeks prior to the start of the experiment with free access to standard diet and tap water. This study was carried out in accordance with the recommendations of the Regulations for the Administration of Affairs Concerning Experimental Animals approved by the State Council of People’s Republic of China. The protocol was approved by the Institutional Animal Care and Use Committee of the Lanzhou Institute of Husbandry and the Pharmaceutical Science of Chinese Academy of Agricultural Science.

**Figure 5:**
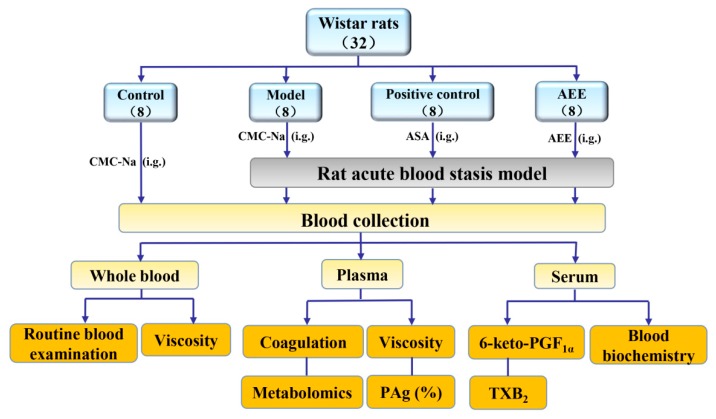
Study design of this experiment. i.g.: intragastric administration; PAg%: platelet aggregation rate.

The design of this study is shown in Figure 5. A total of 32 Wistar rats were randomized to the following four groups (eight rats per group): normal control group (control), blood stasis model group (model), positive control group (ASA) and AEE-pretreated group (AEE). ASA and AEE were suspended in 0.5% CMC-Na. Previous studies have indicated that a treatment of 100 mg/kg ASA could significantly increase blood fluidity in the rat blood stasis model [27]; therefore, an ASA dose of 100 mg/kg was administered to the rats in the ASA group. To compare the results, AEE (180 mg/kg) was administered an equimolar dose of ASA at 0.55 mmol/kg, whereas both the control and model groups were administered 0.5% CMC-Na (30 mg/kg) with a volume that was nearly equal to that of the other groups. All groups were administered their corresponding drugs by gavage seven times with an interval of 12 h between each dose.

### 4.3. Rat Acute Blood Stasis Model

The rat ABS models were established as described in a previous study [27]. Briefly, the rat ABS models were established by placing the rats in ice-cold water (0–4 °C) during the interval between the two injections of adrenaline hydrochloride (Adr). The rats in the control group were subcutaneously injected with normal saline solution (0.9% NaCl, w/v) after the fifth drug administration, whereas rats in the other groups were injected with Adr (0.8 mg/kg). Two hours later, rats from all groups except the control group were placed in ice-cold water for 5 min. Two hours after being placed in this ice bath, all of the other rats were subcutaneously re-injected with Adr (0.8 mg/kg) to establish the rat ABS model; rats in the control group were re-injected with normal saline solution (0.9% NaCl, w/v). Rats were fasted overnight with free access to clean water. In addition, the sixth and seventh drug administrations were continued after performing the model treatment. Adrenaline and stimulation from the ice-water bath could cause peripheral vascular injury, blood vessel contraction, blood shear stress changes and damage to the vascular wall and endothelial cells, which could induce the development of blood stasis. This model simulates a condition of blood stasis that is similar to that seen in humans, and the final pathological changes that are observed in rat blood are similar to those observed in human blood.

### 4.4. Sample Collection

Thirty minutes after the final drug administration, rats were anaesthetized with sodium pentobarbital (40 mg/kg) and blood was collected from the abdominal aorta. A total of 1 mL blood was collected into clean plastic tubes to obtain serum samples after centrifuging for 10 min at 3600 *g*; almost 400 µL serum was used for the blood biochemistry tests, and 100 µL serum was used to detect TXB_2_ and 6-keto-PGF_1α_. Then, 1.8 mL blood was collected into plastic tubes with 3.8% sodium citrate, and the plasma was prepared (3600 *g* for 10 min) for the analysis of the plasma coagulation cascade indicators. A 2-mL blood sample was collected in 3.8% sodium citrate with a blood/anticoagulant ratio of 9:1 for the analysis of platelet aggregation. Additionally, a 3-mL whole blood sample was anticoagulated by the addition of heparin sodium, 2.3 mL was used to prepare the plasma (3600 *g* for 10 min at 4 °C for the plasma viscosity (PV) analysis and metabolomic study), and the remaining sample was used for the whole blood viscosity (WBV) measurement and routine blood examination.

### 4.5. Viscosity and Platelet Aggregation Determination

Six-hundred microliter whole blood and plasma samples were maintained at 37 °C and used to measure the WBV and PV, respectively, with an LBY-N6A rheometer (Precil Co., Beijing, China). The WBV was measured at all of the shear rates (1, 5, 30 and 200 s^−1^), while the PV was measured at shear rates of 30 and 200 s^−1^. Platelet aggregation was measured with a Chrono-log Platelet Aggregometer Model 700 (Chrono-log Corp., City, State, USA). Platelet-rich plasma (PRP) was obtained by centrifugation of citrated whole blood at 200 *g* for 15 min at room temperature, and platelet-poor plasma (PPP) was obtained by an additional centrifugation step at 804 *g* for 10 min. Platelet aggregation was induced by adding 10 μM of adenosine diphosphate (ADP) and 0.5 mM arachidonic acid (AA).

### 4.6. Plasma Anticoagulation Assay

The plasma coagulation cascade indicators, including prothrombin time (PT), fibrinogen content (FIB) and thrombin time (TT) were analyzed. A 2-mL sample of blood that was anticoagulated with 3.8% sodium citrate was examined with detection kits by a blood coagulation analyzer (Sysmex CS-5100 System, Erlangen, Germany).

### 4.7. Haematological Analysis and Blood Biochemistry

Approximately 100 µL blood was measured by a BC-2800vet hematology analyzer (Mindray, Shenzhen, China) for a routine blood examination. Then, the red blood cell (RBC), hemoglobin (HGB), hematocrit (HCT), white blood cell (WBC), percentage of platelet (PCT), platelet (PLT), lymphocyte (Lymph), monocyte count (MONON), neutrophilic granulocyte (Gran), erythrocyte mean corpuscular hemoglobin (MCH), erythrocyte mean corpuscular hemoglobin concentrate (MCHC), erythrocyte mean corpuscular volume (MCV), red blood cell distribution width (RDW), mean platelet volume (MPV), platelet distribution width (PDW) and the percentage of monocyte (Mon%), neutrophilic granulocyte (Gran%) and lymphocyte (Lymph%) values were recorded. Almost 400 µL serum were used to measure the blood biochemistry indexes, including T-BIL, TP, ALB, GLB, ALT, AST, ALP, LDH, BUN, CR, GLU, CK, UA and TG using an XL-640 automatic biochemistry analyzer (Erba, Germany). A 100 µL serum sample was used to measure TXB_2_ and 6-keto-PGF_1α_ levels with the corresponding ELISA kits (Cayman Chemical, Ann Arbor, USA).

### 4.8. Sample Preparation for Metabolomics Study

Before analysis, the plasma samples were thawed at room temperature. The plasma (200 μL) was added into cold methanol (−20 °C, 600 μL), vortex-mixed for 30 s, then centrifuged at 12,000 g at 4 °C for 10 min to precipitate the proteins. After centrifugation, the supernatant was collected and filtered (0.22 μm filter), and 4 μL were injected for MS analysis. The stability and repeatability of the system were validated through quality control (QC) samples, which were prepared by pooling equal volumes of treated plasma. The QC samples were inserted into the analysis sequence.

### 4.9. UPLC-MS Conditions

Chromatographic separations were conducted on an Agilent ZORBAX Eclipse plus C18 RRHD column (2.1 × 150 mm, 1.8 μm) with an Agilent 1290 UPLC system. The column temperature was set at 35 °C, and the post time for column equilibration was 5 min. The column was eluted at a flow rate of 0.25 mL/min, with a mobile phase of (A) 0.1% (*v*/*v*) formic acid in water and (B) acetonitrile with 0.1% formic acid (*v*/*v*). The gradient elution program for the plasma sample was optimized as follows: 0–2 min, 5% B; 2–8 min, 25% B; 8–20 min, 95% B; 20–22 min, 95% B; 22–23 min, 5% B; and 23–25 min, 5% B.

An Agilent 6530 Q-TOF mass spectrometer (Agilent Technologies, CA, USA) was used to perform the MS data acquisition with the electrospray ionization source both in positive (ESI+) and negative (ESI−) ion modes. The ion source parameters were set as follows: nitrogen as drying gas at a flow of 10 L/min at 350 °C; the capillary voltages in ESI+ and ESI− modes were 4.0 and 3.5 KV, respectively; fragment voltage, 135 V; skimmer voltage, 65 V; nebulizer pressure, 45 psig; and acquisition rate, 1 spectra/s. The range of data correction was from 50–1000 m/z in centroid mode.

### 4.10. Multivariate Analysis and Identification of Potential Metabolites

Data analysis was performed as described in our previous report, with minor modifications [55]. The raw MS spectra data (.d) were first converted to a common data format (.mzData) in Mass Hunter Qualitative Analysis software (B.06.00, Santa Clara, CA USA), then the data were analyzed by the XCMS program (V 03.03, La Jolla, CA, USA). The data were then filtered by interquartile ranges and normalized with MetaboAnalyst (V 4.0, Montreal, Quebec, Canada). The normalized data matrix was imported and processed by SIMCA-P V13.0 (Umetrics AB, Malmo, Sweden). Pattern recognition methods, including unsupervised principal component analysis (PCA) and supervised orthogonal partial least squares discriminant analysis (OPLS-DA), were performed in SIMCA. A score plot (S-plot) was used to visualize the results that could reflect differences among different groups. The R^2^ and Q^2^ values were employed to describe the model quality, and the S-plot was used to display the variables that contributed to the classification. A variance importance for projection (VIP) value constructed from the OPLS-DA model and *p*-values were used to select the potential metabolites (VIP > 1.0 and *p* < 0.05).

Metabolite identification was performed based on the accurate mass and mass spectrometric fragmentation patterns. The metabolites were qualified by searching the Human Metabolome Database (HMDB), Massbank and METLIN databases. Biochemical reactions that were involved were searched using the Kyoto Encyclopedia of Genes and Genomes (KEGG). Identified differential metabolites were then analyzed by the MetaboAnalyst (V 4.0, Montreal, Quebec, Canada) for metabolic pathway evaluation.

### 4.11. Statistical Analysis

One-way ANOVA followed by a Duncan’s multiple range test was used for data analysis (SPSS 17.0, IBM, Almond, NY, USA). Data were presented as mean ± standard deviation (SD), and *p*-values less than 0.05 were considered to be statistically significant.

## 5. Conclusions

In Conclusion, ABS rats exhibited damaged blood rheology. Treatment with either ASA or AEE could mitigate blood stasis and was characterized by the ability to modulate WBV, PV, blood coagulation parameters, platelet count, platelet aggregation, CR, LDH and the levels of TXA_2_ and 6-keto-PGF_1α_. AEE and ASA treatments had similar effects on these indicators in ABS rats. Differences in plasma metabolite profiles and abundance between AEE and ASA groups were observed. Nineteen potential metabolites that were related to the metabolism of amino acid, fatty acid, energy and glycerophospholipid were identified and determined to be regulated by AEE treatment, which could be used to explain the possible mechanism by which AEE attenuates blood stasis. The current study also indicated that LC-MS-based metabolomics is a valuable approach for investigating the efficacy of the drug.

## Figures and Tables

**Table 1:** Plasma coagulation cascade indicators of different groups.

Groups	Plasma Coagulation Parameters		
	PT (s)	TT (s)	FIB (mg/dL)
Control	8.11 ± 0.21	38.26 ± 1.23 ^##^	1.63 ± 0.17 ^##^
Model	8.65 ± 0.22	29.94 ± 2.73	3.85 ± 0.12
ASA	11.56 ± 1.77 ^##^	31.93 ± 1.07 ^#^	3.69 ± 0.13 ^#^
AEE	10.86 ± 0.46 ^##^	31.71 ± 0.55 ^#^	3.21 ± 0.19 ^#▲^

PT: Prothrombin time; TT: thrombin time; FIB: fibrinogen. Data are presented as means ± SD; *n* = 8; ^#^ *p* < 0.05, ^##^ *p* < 0.01, compared with the model group; ^▲^ *p* < 0.05, compared with the ASA group.

**Table 2:** Effects of AEE on haematological analysis (**A**) and blood biochemistry (**B**).

Variable	Unit	Control	Model	ASA	AEE
A: Haematological Analysis					
WBC	10^9^/L	4.19 ± 0.63 ^##^	8.11 ± 0.76	8.6 ± 2.4	10.13 ± 1.28 ^##^
Lymph	10^9^/L	2.84 ± 0.55 ^##^	1.59 ± 0.34	1.64 ± 0.58	1.93 ± 0.69
MONON	10^9^/L	0.1 ± 0.01 ^##^	0.23 ± 0.07	0.21 ± 0.10	0.28 ± 0.07
Gran	10^9^/L	1.25 ± 0.29 ^##^	6.3 ± 0.48	6.75 ± 1.83	7.93 ± 0.94 ^##^
Lymph	%	65.26 ± 5.54 ^##^	19.56 ± 2.8	18.7 ± 3.21	19.2 ± 3.44
Mon	%	2.76 ± 0.38	2.79 ± 0.49	2.63 ± 0.39	2.75 ± 0.30
Gran	%	30.01 ± 5.68 ^##^	77.65 ± 3.24	78.68 ± 3.25	78.05 ± 3.57
RBC	10^12^/L	7.76 ± 0.40 ^#^	8.46± 1.04	7.91 ± 0.17	8.42 ± 0.54
HGB	g/L	145.5 ± 8.44 ^#^	160.4 ± 23.90	147.5 ± 4.47	155 ± 5.03
HCT	%	44.05 ± 2.21	47.59 ± 6.340	44.16 ± 0.96	46.84 ±1.39
MCV	fL	56.88 ± 0.37	56.25 ± 1.08	55.89 ± 0.52	56.55 ± 0.66
MCH	pg	18.71 ± 0.25	18.85 ± 0.56	18.59 ± 0.37	18.64 ± 0.28
MCHC	g/L	329.6 ± 4.57 ^#^	335.9 ± 5.59	333.4 ± 4.57	330.5 ± 6.07 ^#^
RDW	%	10.99 ± 0.51	10.83 ± 0.46	10.83 ± 0.41	10.95 ± 0.50
PLT	10^9^/L	833.9 ± 68.51 ^##^	440.9 ± 140.8	600.9 ± 24.2 ^##^	678.4 ± 50.0 ^##^
MPV	fL	5.75 ± 0.26	5.7 ± 0.21	5.41 ± 0.11 ^##^	5.9 ± 0.10
PDW	%	16.26 ± 0.13 ^#^	16.46 ± 0.12	16.39 ± 0.19	16.43 ± 0.14
PCT	%	0.48 ± 0.03 ^#^	0.25 ± 0.08	0.33 ± 0.01 ^##^	0.38 ± 0.03 ^##▲^
B: Blood Biochemistry					
T-BIL	μM	1.66 ± 0.30	1.79 ± 0.64	0.60 ± 0.11 ^##^	0.60 ± 0.14 ^##^
TP	g/L	64.73 ± 2.29	63.03 ± 1.19	60.11 ± 2.46 ^##^	60.78 ± 1.56 ^##^
ALB	g/L	29.16 ± 0.68 ^##^	27.55 ± 1.08	27.35 ± 0.83	27.50 ± 1.09
GLB	g/L	35.56 ± 1.99	35.48 ± 1.06	32.76 ± 1.95 ^##^	33.28 ± 1.08 ^##^
ALB/GLB		0.84 ± 0.05 ^#^	0.78 ± 0.05	0.84 ± 0.05 ^#^	0.81 ± 0.04
ALT	U/L	55.78 ± 7.71 ^##^	203.7 ± 40.22	414.3 ± 117.3 ^##^	358.0 ± 66.87 ^##^
AST	U/L	170.13 ± 10.16 ^##^	818.5 ± 228.9	1155 ± 212 ^##^	1223 ± 227 ^##^
AST/ALT		3.13 ± 0.47 ^##^	3.99 ± 0.49	2.89 ± 0.45 ^##^	3.41 ± 0.37 ^#▲^
ALP	U/L	95.75 ± 17.69 ^#^	84.00 ± 6.65	84.25 ± 5.09	83.50 ± 3.89
LDH	U/L	2513 ± 173.9 ^##^	2872 ± 250	2605.4 ± 143.9 ^#^	2653 ± 212.1 ^#^
CK	U/L	987.6 ± 89.02	1099 ± 450.8	1831 ± 480 ^##^	2169 ± 522.2 ^##^
BUN	mM	7.39 ± 0.34 ^##^	8.68 ± 0.79	8.76 ± 1.12	10.27 ± 0.71 ^##^
CR	μM	40.11 ± 1.25 ^##^	51.36 ± 8.32	40.01 ± 4.06 ^##^	46.11 ± 3.58 ^#▲^
UA	μM	0.06 ± 0.02	0.08 ± 002	0.17 ± 0.12^##^	0.08 ± 0.02^▲^
GLU	mM	5.98 ± 0.66 ^##^	3.88 ± 0.62	3.96 ± 0.72	3.68 ± 0.35
TG	mM	0.76 ± 0.16	0.83 ± 0.10	1.15 ± 0.19 ^##^	1.17 ± 0.06 ^##^

RBC: red blood cell; HGB: hemoglobin; HCT: hematocrit; WBC: white blood cell; PCT: percentage of platelet; PLT: platelet; Lymph: lymphocyte; MONON: monocyte count; Gran: neutrophilic granulocyte; MCH: erythrocyte mean corpuscular hemoglobin; MCHC: erythrocyte mean corpuscular hemoglobin concentrate; MCV: erythrocyte mean corpuscular volume; RDW: red blood cell distribution width; MPV: mean platelet volume; PDW: platelet distribution width; Mon%: the percentage of monocyte; Gran%: neutrophilic granulocyte; Lymph%: percentage of lymphocyte; T-BIL: the total bilirubin; TP: total protein; ALB: albumin; GLB: globulin; ALT: alanine transaminase; AST: aspartate aminotransferase; ALP: alkaline phosphatase; LDH: lactate dehydrogenase; BUN: blood urea nitrogen; CR: creatinine; GLU: glucose; CK: creatine kinase; UA: uric acid; TG: triglyceride.^.#^ *p* < 0.05, ^##^ *p* < 0.01, compared with the model group; ^▲^ *p* < 0.05, compared with the ASA group. Data are presented as means ± SD; *n* = 8.

**Table 3:** Identification results for the candidate metabolites in the plasma of rats with blood stasis.

No.	SM	Formula	*m*/*z*	RT	Metabolite	Fold Change		
						M/C	ASA/M	AEE/M
1	ESI+	C_30_H_50_NO_7_P	568.3406	17.68	LysoPC (22:6)	0.47 **	0.84	1.10
2	ESI+	C_26_H_48_NO_7_P	518.3243	16.91	LysoPC (18:3)	0.29 **	1.71 **	2.43 **
3	ESI+	C_4_H_9_N_3_O_2_	132.0769	1.36	Creatine	2.40 **	0.70 *	0.92
4	ESI+	C_23_H_48_NO_7_P	482.3261	17.91	LysoPC (15:0)	0.53 **	0.68 **	1.38 **
5	ESI+	C_9_H_17_NO_4_	204.1225	2.03	Acetylcarnitine	1.75 **	0.62 **	0.61 *
6	ESI+	C_28_H_50_NO_7_P	544.3412	17.85	LysoPC (20:4)	0.47 **	0.80 *	1.20 *
7	ESI+	C_23_H_45_NO_4_	400.3426	22.44	Palmitoylcarnitine	2.36 **	1.08	0.60 **
8	ESI+	C_24_H_50_NO_7_P	496.3411	19.78	LysoPC (16:0)	0.63 **	0.88 *	1.54 **
9	ESI+	C_26_H_50_NO_7_P	520.3410	18.06	LysoPC (18:2)	0.54 **	0.89	1.61 **
10	ESI+	C_9_H_11_NO_2_	166.0858	3.65	Phenylalanine	1.24 **	1.05	0.77 **
11	ESI+	C_24_H_48_NO_7_P	494.3246	17.29	LysoPC (16:1)	0.56 **	1.16	1.48 **
12	ESI+	C_6_H_13_NO_2_	132.1020	2.16	Isoleucine	1.36 **	0.65 **	0.33 **
13	ESI+	C_5_H_11_NO_2_	118.0862	1.95	Valine	1.21	0.66 **	0.60 **
14	ESI-	C_18_H_32_O_2_	279.2328	22.01	Linoleic acid	2.19 *	0.62	0.40 *
15	ESI-	C_18_H_34_O_2_	281.2483	23.43	Oleic acid	2.39 *	0.69	0.43 *
16	ESI-	C_22_H_32_O_2_	327.2328	21.38	Docosahexaenoic acid	1.50 **	0.59 **	0.42 **
17	ESI-	C_11_H_12_N_2_O_2_	203.0817	5.99	Tryptophan	1.09	0.56 **	0.49 **
18	ESI-	C_20_H_32_O_2_	303.2327	21.69	Copalic acid	1.29	0.59 **	0.52 **
19	ESI-	C1_6_H_32_O_2_	255.2326	23.21	Palmitic acid	2.1 5 **	0.59	0.34 **

SM: scan mode; RT: retention time; M/C: model vs control; ASA/M: ASA vs model; AEE/M: AEE vs model. * *p* < 0.05, ** *p* < 0.01.

## Supplementary Materials

The following are available online. Figure S1: Typical UPLC-Q-TOF/MS total ion chromatograms of rat plasma in each condition in positive (A) and negative ion modes (B); Figure S2: PCA score plots of plasma analyzed by UPLC-TOF/MS in positive and negative modes; Figure S3: S-plots of the corresponding OPLS-DA models in positive and negative ion modes; Figure S4: Heatmap and cluster analysis of the significantly changed metabolites in different groups; Table S1: Comparison of the metabolites relative intensity in different group. Table S2: Pathway analysis results from MetaboAnalyst.
