# Supplementary material for: UPLC-Q-TOF/MS-Based Plasma Metabolomics to Evaluate the Effects of Aspirin Eugenol Ester on Blood Stasis in Rats
Source: Molecules. 2019 Jun 27;24(13):2380. doi: 10.3390/molecules24132380 (PMC6651160; doi:10.3390/molecules24132380)
Supplement: Supplementary file 1 [file molecules-24-02380-s001.pdf]

Figure S1 Typical UPLC-Q-TOF/MS total ion chromatograms of rat plasma in each condition in positive (A) and negative ion modes (B).

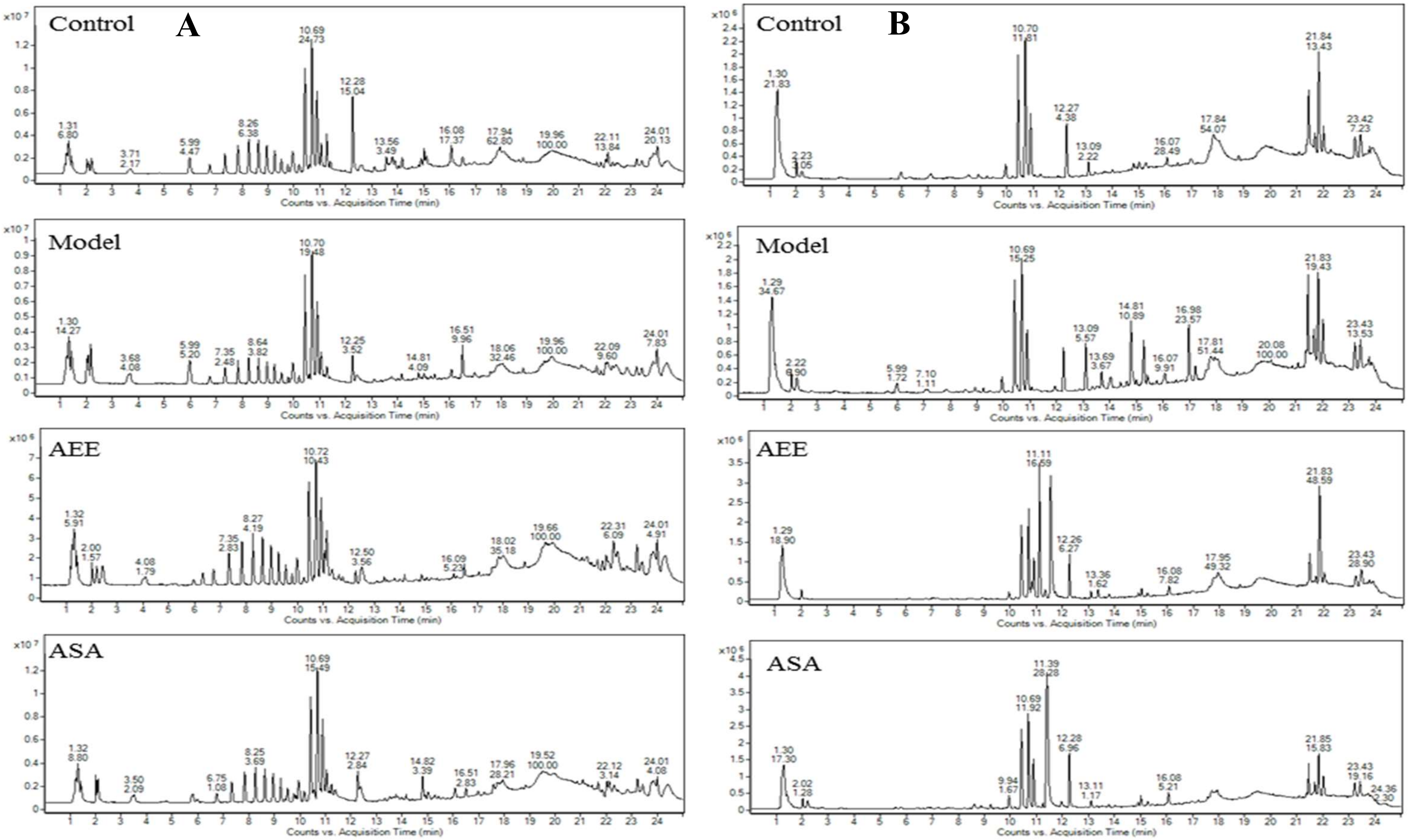

The x-axis was the retention time (min) and the y-axis was the intensity. Retention time (min) and relative intensity (%) were labeled on the peaks (up and below, respectively).

Figure S2 PCA score plots of plasma analyzed by UPLC-TOF/MS in positive and negative modes.

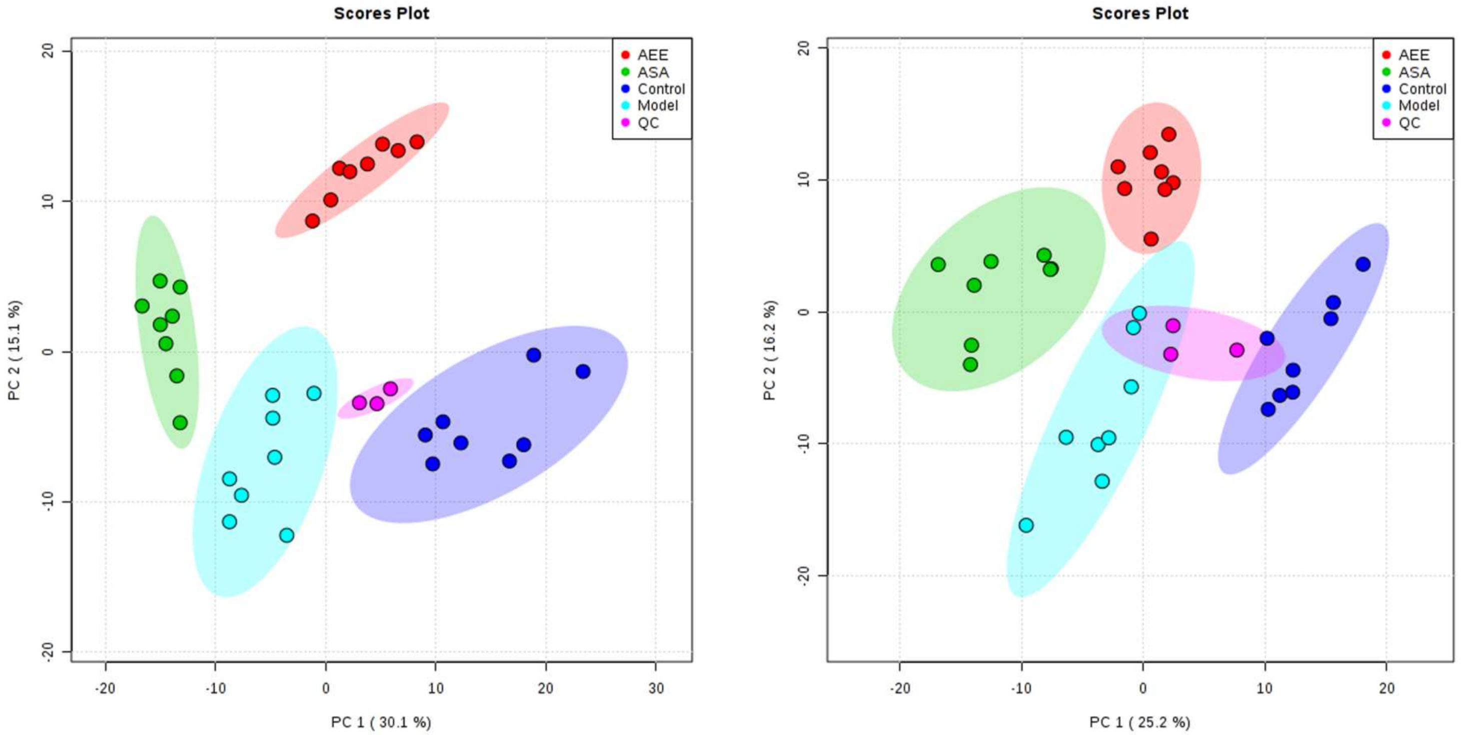

PCA score plots of all the samples in the study. ESI+: $R^2X = 0.452$, ESI-: $R^2X = 0.414$. All the QC samples were clustered tightly, indicating the analysis method was robust with good repeatability and stability.

Figure S3 OPLS-DA score plots of the plasma samples in AEE and ASA groups.

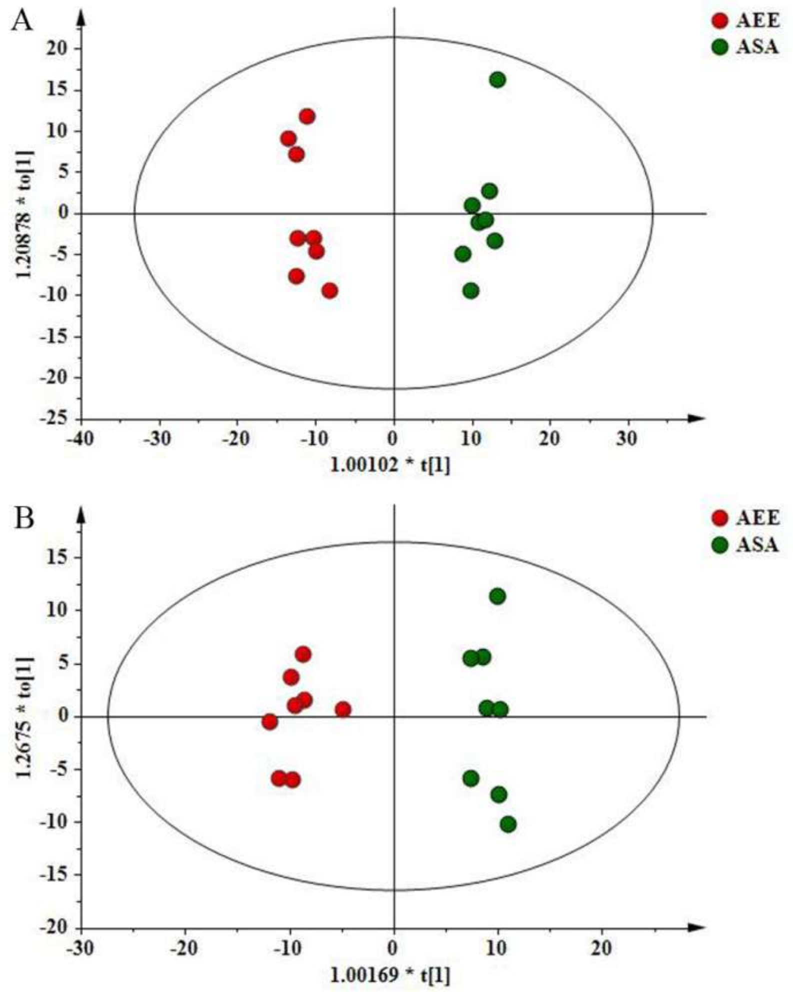

OPLS-DA analysis was performed between AEE and ASA groups. A: positive ion mode, $R^2X = 0.551$, $R^2Y = 0.981$ and $Q^2 = 0.968$. B: negative ion mode, $R^2X = 0.418$, $R^2Y = 0.969$ and $Q^2 = 0.89$.

Figure S4 S-plots of the corresponding OPLS-DA models in positive and negative ion modes.

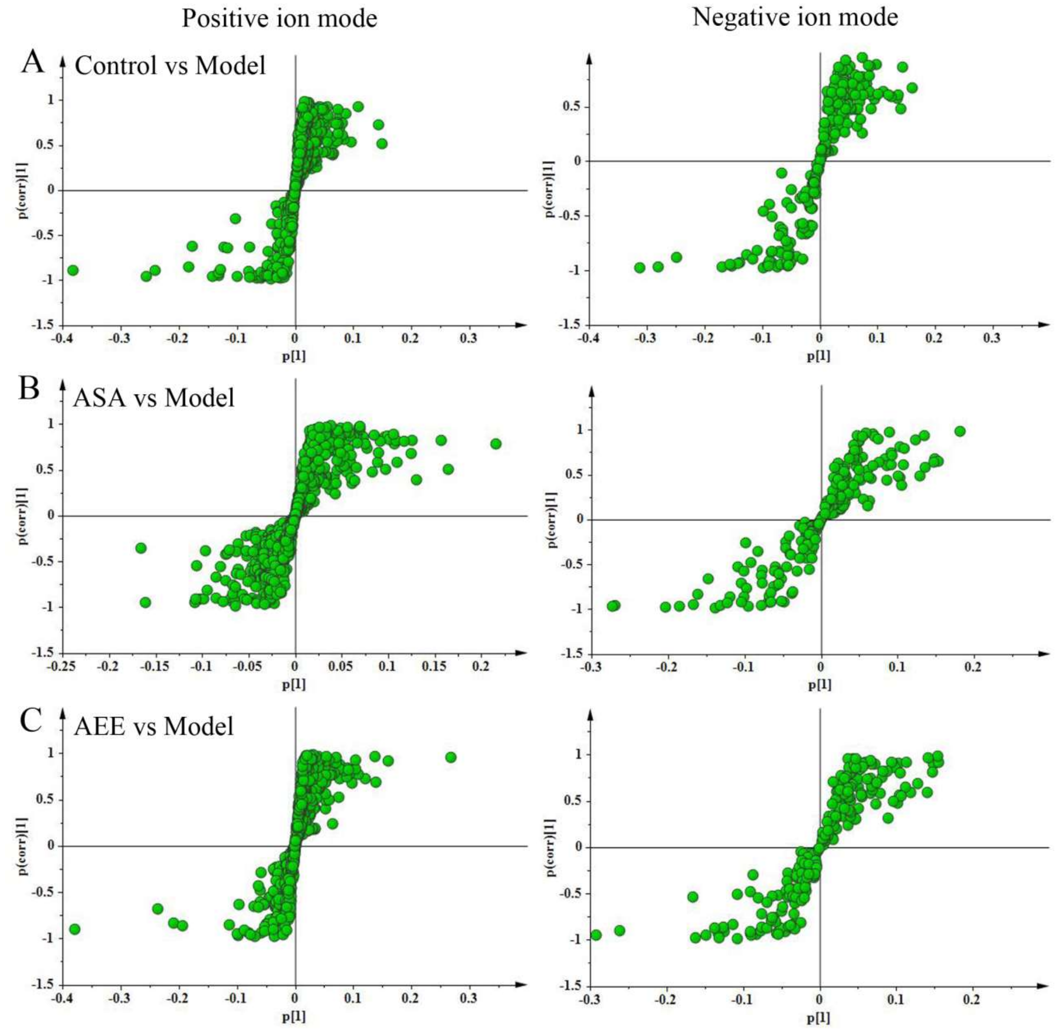

S-plot was applied to visualize the variable influence, which combines the covariance (x-axis) and correlation (y-axis) loading profiles resulting from OPLS-DA models.

Figure S5 Heatmap and cluster analysis of the significantly changed metabolites in different groups.

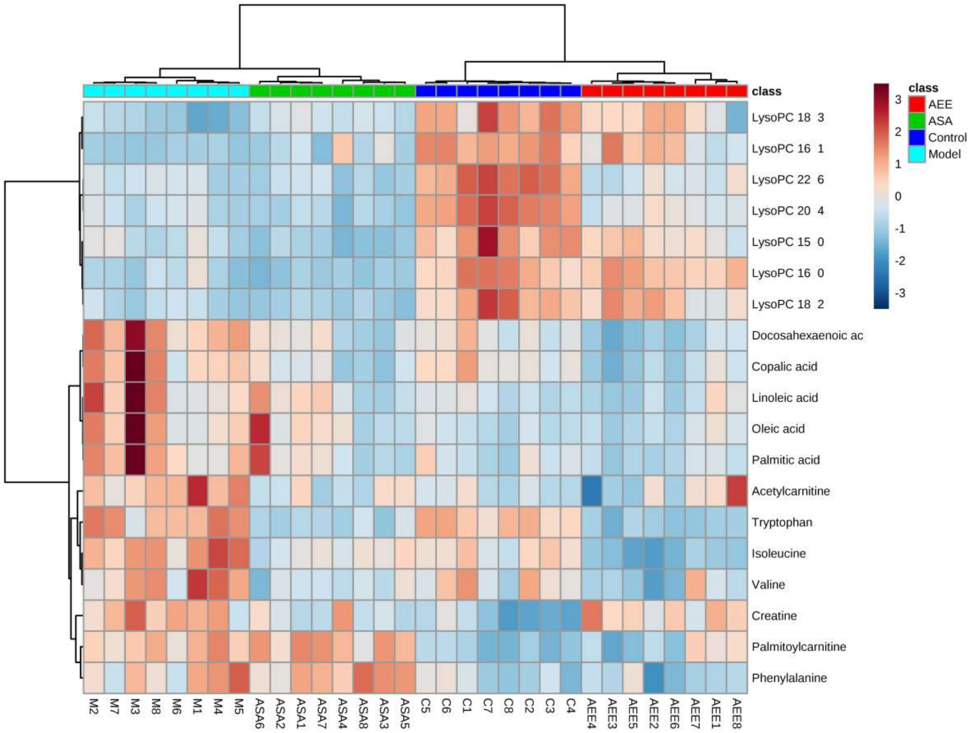

C: samples in control group; M: samples in HFD group; AEE: samples in AEE group; ASA: samples in aspirin group.

Table S1 Comparison of the metabolites relative intensity in different group.

No.	Metabolite	Relative intensity			
		Control	Model	ASA	AEE
1	LysoPC (22:6)	9.09±1.08$^{\#\#}$	4.24±0.71	3.58±0.75	4.65±0.78$^{\blacktriangle}$
2	LysoPC (18:3)	0.58±0.12$^{\#\#}$	0.17±0.07	0.28±0.03$^{\#\#}$	0.4±0.14$^{\#\#}$
3	Creatine	1.39±0.65$^{\#\#}$	3.34±0.71	2.35±0.69$^{\#}$	3.06±0.61
4	LysoPC (15:0)	1.57±0.34$^{\#\#}$	0.83±0.16	0.56±0.10$^{\#\#}$	1.14±0.18$^{\#\#\blacktriangle\blacktriangle}$
5	Acetylcarnitine	0.90±0.17$^{\#\#}$	1.58±0.35	0.98±0.25$^{\#\#}$	0.97±0.64$^{\#}$
6	LysoPC (20:4)	34.88±3.55$^{\#\#}$	16.45±2.64	13.22±2.27$^{\#}$	19.80±2.21$^{\#\blacktriangle\blacktriangle}$
7	Palmitoylcarnitine	2.52±0.65$^{\#\#}$	5.97±0.95	6.44±1.14	3.55±1.56$^{\#\#\blacktriangle\blacktriangle}$
8	LysoPC (16:0)	117.87±15.92$^{\#\#}$	74.19±10.18	65.48±4.41$^{\#}$	113.89±9.21$^{\#\#\blacktriangle\blacktriangle}$
9	LysoPC (18:2)	37.98±7.04$^{\#\#}$	20.57±2.74	18.24±1.72	33.07±6.09$^{\#\#\blacktriangle\blacktriangle}$
10	Phenylalanine	8.99±1.00$^{\#\#}$	11.15±1.57	11.74±1.14	8.57±1.09$^{\#\#\blacktriangle\blacktriangle}$
11	LysoPC (16:1)	2.13±0.16$^{\#\#}$	1.19±0.05	1.38±0.26	1.77±0.33$^{\#\#\blacktriangle}$
12	Isoleucine	19.65±3.16$^{\#\#}$	26.81±5.08	17.5±2.59$^{\#\#}$	8.86±2.34$^{\#\#\blacktriangle\blacktriangle}$
13	Valine	1.75±0.38	2.12±0.46	1.4±0.21$^{\#\#}$	1.28±0.41$^{\#\#}$
14	Linoleic acid	2.86±0.67$^{\#}$	6.26±3.22	3.87±1.17	2.51±1.3$^{\#}$
15	Oleic acid	0.99±0.24$^{\#}$	2.36±1.16	1.62±1.03	1.01±0.35$^{\#}$
16	Docosahexaenoic acid	2.53±0.46$^{\#\#}$	3.81±0.92	2.24±0.55$^{\#\#}$	1.59±0.43$^{\#\#\blacktriangle}$
17	Tryptophan	5.02±0.53	5.47±0.85	3.06±0.41$^{\#\#}$	2.67±0.31$^{\#\#}$
18	Copalic acid	2.73±0.45	3.52±1.07	2.09±0.47$^{\#\#}$	1.82±0.45$^{\#\#}$
19	Palmitic acid	0.83±0.36$^{\#\#}$	1.78±0.80	1.05±0.71	0.61±0.21$^{\#\#}$

Data was expressed as mean ± SD; n = 8; $^{\#}$ $P < 0.05$, $^{\#\#}P < 0.01$ compared with model group. $^{\blacktriangle}P < 0.05$, $^{\blacktriangle\blacktriangle}P < 0.01$, compared with the ASA group.

Table S2 Pathway analysis results from MetaboAnalyst.

	Total	Expected	Hits	Raw P	Holm adjust	FDR	Impact
Biosynthesis of unsaturated fatty acids	42	0.35568	4	0.000278	0.022828	0.022828	0
Aminoacyl-tRNA biosynthesis	69	0.58433	4	0.001897	0.15369	0.077793	0.08
Valine, leucine and isoleucine biosynthesis	11	0.093155	2	0.003468	0.27742	0.094786	0.28572
Phenylalanine, tyrosine and tryptophan biosynthesis	4	0.033874	1	0.033482	1	0.5108	0.5
Valine, leucine and isoleucine degradation	38	0.32181	2	0.039027	1	0.5108	0.03922
Fatty acid metabolism	39	0.33028	2	0.040943	1	0.5108	0.02083
Fatty acid biosynthesis	43	0.36415	2	0.048964	1	0.5108	0
Linoleic acid metabolism	6	0.050812	1	0.049834	1	0.5108	0.5
Phenylalanine metabolism	11	0.093155	1	0.089612	1	0.81646	0.4
Pantothenate and CoA biosynthesis	15	0.12703	1	0.12033	1	0.9867	0.07692
Fatty acid elongation in mitochondria	27	0.22865	1	0.20687	1	1	0
Glycerophospholipid metabolism	30	0.25406	1	0.22725	1	1	0.05263
Glycine, serine and threonine metabolism	31	0.26253	1	0.23393	1	1	0
Tryptophan metabolism	40	0.33874	1	0.29176	1	1	0.13514
Arginine and proline metabolism	44	0.37262	1	0.31616	1	1	0.02041

Total: The total number of compounds in the pathways; the hits are the actually matched number from the upload data; the raw p is the original p value calculated from the enrichment analysis; the impact is the pathway impact value calculated from pathway analysis.
